# Treatments for Depression and the Risk of Dementia: A 23‐Year Cohort Study Using Akrivia Health Secondary Mental Healthcare Data

**DOI:** 10.1002/alz70861_108228

**Published:** 2025-12-23

**Authors:** Panagiota Kontari, Maxime Taquet, William Pettersson‐Yeo, Judith Rose Harrison, Ana Todorovic, Benjamin Fell

**Affiliations:** ^1^ Akrivia Health, Oxford, NA UK; ^2^ University of Oxford, Oxford Health NHS Foundation Trust, Oxford UK; ^3^ Oxford University, Oxford, NA UK; ^4^ Translational and Clinical Research Institute, Faculty of Medical Sciences, Newcastle University, Newcastle upon Tyne, England UK; ^5^ Akrivia Health, Oxford UK

## Abstract

**Background:**

Depression has been suggested as a modifiable risk factor for dementia. However, the impact of depression treatments on dementia risk remains unclear. This study assesses the association between exposure to different antidepressants and psychotherapy, and the risk of dementia.

**Method:**

Using Akrivia Health’s mental healthcare database (anonymised electronic health records for 5.1 million patients in the UK), we identified all patients with major depressive disorder diagnosed between January 2000 and September 2022, aged 40 or older at diagnosis, without co‐morbid bipolar disorder and/or schizophrenia, and dementia‐free two years post‐depression diagnosis. Patients were categorised into eight groups based on treatment type: psychotherapy only, tricyclic antidepressants (TCAs), monoamine oxidase inhibitors (MAOIs), selective serotonin re‐uptake inhibitors (SSRIs), serotonin and noradrenaline re‐uptake inhibitors (SNRIs), other antidepressants, and combined pharmacotherapy (≥ 2 antidepressants), with untreated patients as reference. Cox proportional hazards regression models estimated hazard ratios (HR) for dementia, adjusted for age, gender, and ethnicity.

**Result:**

A total of 56,423 individuals were identified (6,904 developed dementia during follow‐up, 59% female, mean[SD] age: 67.6 [10.7]). Compared to untreated patients, all patients treated with antidepressants, but not those treated with psychotherapy only, showed a significantly greater risk of dementia, especially MAOIs (HR=2.15,95% confidence interval [CI] 1.39−3.34) and TCAs (HR=1.82, 95% CI 1.52−2.18). TCAs conferred a significantly greater risk than SSRIs (HR=1.39, 95% CI 1.29−1.50).

**Conclusion:**

This study showed that antidepressants, especially MAOIs and TCAs, are linked to an increased risk of dementia. The increased risk may be due to anticholinergic effects of antidepressants as well as residual confounding due to depression severity. Further research is needed to understand the underlying mechanisms and help clinicians balance the benefits of depression treatment with the risk of dementia.

